# The effect of a daily quiz (TOPday) on self-confidence, enthusiasm, and test results for biomechanics

**DOI:** 10.1007/s40037-013-0096-6

**Published:** 2013-11-28

**Authors:** Esther Tanck, Martijn F. H. Maessen, Gerjon Hannink, Sascha M. H. F. van Kuppeveld, Sanneke Bolhuis, Jan G. M. Kooloos

**Affiliations:** 1 Orthopaedic Research Laboratory, Radboud university medical center, PO Box 9101, 6500 HB Nijmegen, the Netherlands; 2 Department for Evaluation, Quality and Development of Medical Education, Radboud university medical center, Nijmegen, the Netherlands; 3 Department of Anatomy, Radboud university medical center, Nijmegen, the Netherlands

**Keywords:** Daily quiz, Biomechanics, Confidence, Enthusiasm, Education, Test results

## Abstract

Many students in Biomedical Sciences have difficulty understanding biomechanics. In a second-year course, biomechanics is taught in the first week and examined at the end of the fourth week. Knowledge is retained longer if the subject material is repeated. However, how does one encourage students to repeat the subject matter? For this study, we developed ‘two opportunities to practice per day (TOPday)’, consisting of multiple-choice questions on biomechanics with immediate feedback, which were sent via e-mail. We investigated the effect of TOPday on self-confidence, enthusiasm, and test results for biomechanics. All second-year students (*n* = 95) received a TOPday of biomechanics on every regular course day with increasing difficulty during the course. At the end of the course, a non-anonymous questionnaire was conducted. The students were asked how many TOPday questions they completed (0–6 questions [group A]; 7–18 questions [group B]; 19–24 questions [group C]). Other questions included the appreciation for TOPday, and increase (no/yes) in self-confidence and enthusiasm for biomechanics. Seventy-eight students participated in the examination and completed the questionnaire. The appreciation for TOPday in group A (*n* = 14), B (*n* = 23) and C (*n* = 41) was 7.0 (95 % CI 6.5–7.5), 7.4 (95 % CI 7.0–7.8), and 7.9 (95 % CI 7.6–8.1), respectively (*p* < 0.01 between A and C). Of the students who actively participated (B and C), 91 and 80 % reported an increase in their self-confidence and enthusiasm, respectively, for biomechanics due to TOPday. In addition, they had a higher test result for biomechanics (*p* < 0.01) compared with those who did not actively participate (A). In conclusion, the teaching method ‘TOPday’ seems an effective way to encourage students to repeat the subject material, with the extra advantage that students are stimulated to keep on practising for the examination. The appreciation was high and there was a positive association between active participation, on the one hand, and self-confidence, enthusiasm, and test results for biomechanics on the other.

## Introduction

It is important to develop a stimulating environment for both students and teachers to improve the quality of medical education. In order to develop such a stimulating environment students should be actively involved in their own education [1], for example through the availability of an interactive learning environment. It was demonstrated that repeated testing results in long-term retention of information, while repeated studying per se did not [2]. In addition, stimulating students to actively repeat subject matter via spaced education has been shown to improve the retention of knowledge [3, 4]. Spaced education combines two principle psychology research findings, i.e. temporal distribution of learning (spacing effect) and retrieval practice (testing effect) [5–8]. With spaced education, the subject matter is presented and repeated over spaced intervals, which enhances an effective retention of knowledge. The purpose of testing is not only to measure the students’ level of knowledge, but also to retain the knowledge for a longer period of time.

Previous studies demonstrated that many students have difficulties learning and understanding basic Newtonian (classical) (bio)mechanics [9, 10]. In a second-year course of Biomedical Sciences at the Radboud university medical center (Radboudumc), biomechanics is taught in the first week and examined at the end of the fourth week. Two challenges related to the method of education are present: (1) it is not obligatory to complete assignments or attend (interactive) lectures; (2) the subject matter is not repeated during the course. According to the student involvement theory [1], students learn more and their personal development improves if they are more actively involved. In addition, it is quite likely that they will be more motivated, learn more, and gain higher test scores when active participation takes place. The challenge is, therefore, to develop a method that encourages students to be more actively involved with the subject matter.

The use of E-learning (web-based learning, online learning, etc.) creates new opportunities to actively involve students with the subject matter [11]. For this study, we combined spaced education with E-learning by designing ‘two opportunities to practice per day (TOPday)’, consisting of multiple-choice questions on biomechanics with immediate feedback, which were sent via e-mail. The purpose of our study was to investigate the effect of TOPday on self-confidence, enthusiasm, and test results for biomechanics.

## Methods

### Participants and organisation of TOPday during the course

**Fig. 1:**
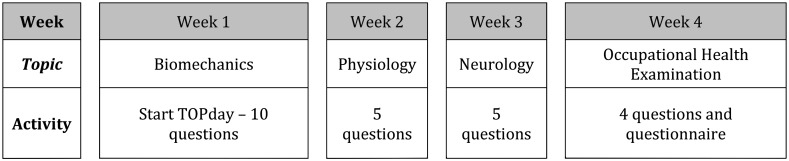
Course organization and the incorporation of TOPday on biomechanics

Many students in Biomedical Sciences of the Radboudumc in the Netherlands have difficulty understanding biomechanics. Biomechanics is taught in a Dutch course, for Dutch students, entitled: ‘Physical Factors’. It is a second-year course for all students doing their Bachelor in Biomedical Sciences. In this course, the students learn basic methods, and work on subjects which are related to Clinical Human Movement Sciences. Basic knowledge about biomechanics is necessary to calculate, for example, joint forces during a certain physical activity. Biomechanics is taught in the first week and examined at the end of the fourth week. During the first week, students could attend one lecture about biomechanics and were asked to do three self-study assignments (SSAs) involving static equilibrium. After each SSA, students could join an interactive lecture where the assignment was discussed with the lecturer. In between, there were two group assignments in which biomechanical problems were solved. During week two, three, and four, students were educated on different topics related to Clinical Human Movement Sciences such as exercise physiology, and neurology (Fig 1).

**Fig. 2:**
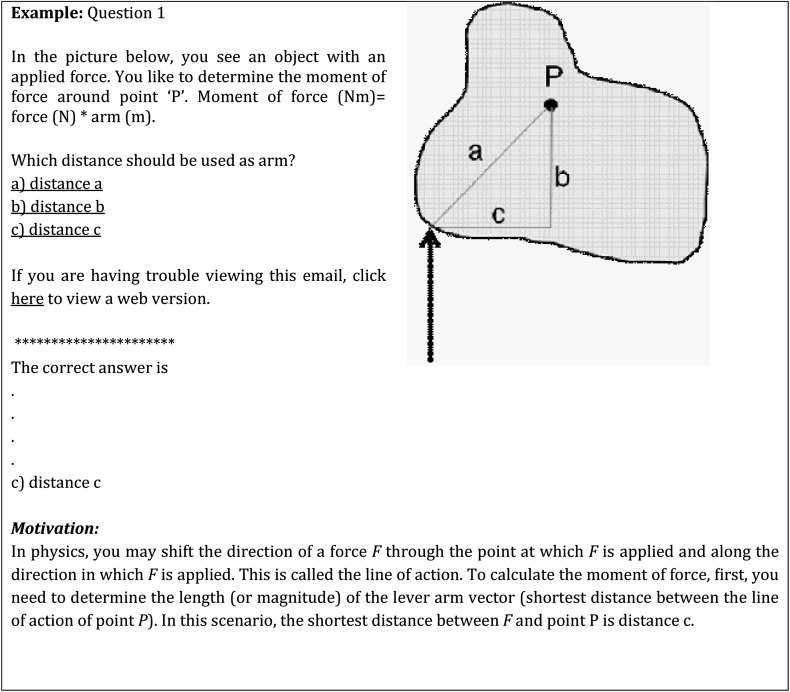
Example of a TOPday question

During the course, all second-year students (*n* = 95) received a TOPday (Fig. 2) of biomechanics on every regular course day. The TOPday consisted of one or two multiple-choice questions with increasing difficulty during the course. By clicking an answer, the students immediately received feedback. In total, 24 TOPday questions were sent to the students via email (Fig. 1). Whether or not the students participated or correctly answered the questions was not automatically checked for. 1 day before the end of the course, a non-anonymous questionnaire was conducted, with questions related to, for example, participation, motivation and appreciation (see Table 1 in Appendix for the questions and answer options; see also paragraph *Outcome measures* and statistical analysis). On the final day of the course the students performed the course examination. This overall exam existed of seven open questions, each consisting of sub-questions. One of the questions concerned biomechanics and its result accounted for 25 % of the final examination score.

**Table 1:**
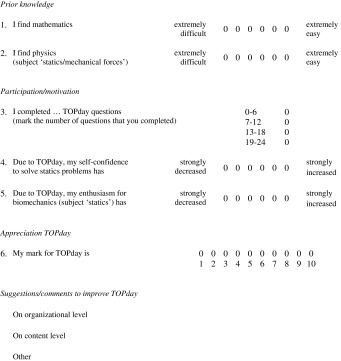
Translated questionnaire

### Ethical considerations

During the introduction lecture on the first day of the course, students were informed about the procedures and purpose of TOPday. All students received the information through email as well. It was stated that participation was voluntary, and that they could unsubscribe from TOPday at any time. They were assured that completing the questionnaire would not affect or have influence on their course results.

In our opinion, participation in TOPday did not have any negative consequences for the students, since they were given the opportunity to voluntarily practice biomechanics on top of their regular classes and assignments. Privacy and confidentiality were guaranteed and the results of this study were only accessible for the researchers involved. The non-anonymous questionnaire was linked to student number, which was subsequently linked to the results of the final exam. Ethical principles of the World Medical Association Declaration of Helsinki were taken into account during the development of the present study.

### Outcome measures and statistical analysis

The following outcome measures were obtained via the non-anonymous questionnaire. (1) Increase (no/yes) in enthusiasm and self-confidence for biomechanics due to TOPday. The variables ‘enthusiasm’ and ‘self-confidence’ were split into no/yes at the midpoint of the scale (range ‘strongly decreased’ to ‘strongly increased’, see Appendix). (2) Number of TOPday questions completed. The four answer options were categorized into low (0–6 questions, group A), medium (7–18 questions, group B), and high participation (19–24 questions, group C). (3) Prior knowledge (−/+) concerning mathematics and physics. The variables were split into −/+ at the midpoint of the scale (range ‘extremely difficult’ to ‘extremely easy’, see Appendix). (4) The students’ appreciation for TOPday (scale 0–10). (5) The total test score for the final course examination, and (6) the subscore for biomechanics only (both scale 0–10) were registered.

Only data from students who participated in both the final examination and completed the questionnaire were analyzed. Test results and final appreciation versus the number of questions completed were analyzed using one-way ANOVA and Bonferroni post hoc tests. Self-confidence and enthusiasm were analyzed using descriptive statistics in the groups of students who actively participated (group B and C).

## Results

### Participation rate and number of questions completed

Seventy-eight out of 95 students (82 %) completed the questionnaire and participated in the final examination. Fourteen students (18 %) completed 0–6 questions (group A), 23 students (29 %) completed 7–18 questions (group B), and 41 students (53 %) completed 19–24 questions (group C).

Seventeen students were not included in our study: seven students missed the exam, hence no test results were available, and the other ten students did not complete the questionnaire (subscore biomechanics 5.0 (95 % CI 2.8–7.2) and overall examination result 4.6 (95 % CI 3.0–6.3)).

### Appreciation

**Fig. 3:**
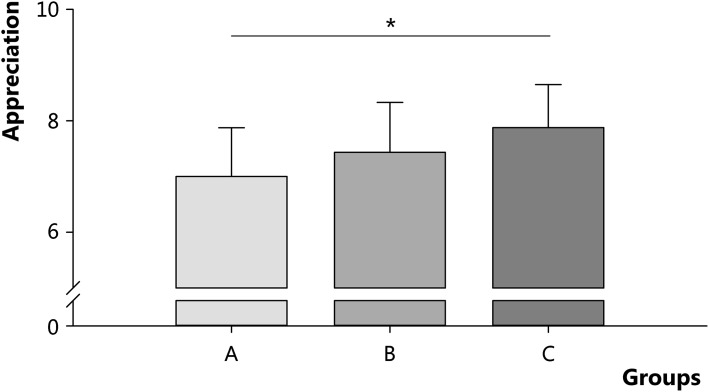
Appreciation for TOPday by students in group A (*N* = 14), B (*N* = 23), and C (*N* = 41). Students in group A, B, and C completed 0–6, 7–18, and 19–24 questions, respectively. Data are presented as mean ± SD per group; * *p* < 0.01

The 78 (35 male and 43 female) students who participated in the examination and completed the questionnaire highly appreciated TOPday. Overall, the final score for TOPday was 7.6 (95 % CI 7.4–7.8). The appreciation was 7.0 (95 % CI 6.5–7.5), 7.4 (95 % CI 7.0–7.8), and 7.9 (95 % CI 7.6–8.1) for students in group A, B, and C, respectively (Fig. 3). Students in group C appreciated TOPday significantly more in comparison with students in group A (*p* < 0.01).

### Self-confidence and enthusiasm

Fifty-eight of the 64 students (91 %) who actively participated (Groups B and C) showed an increase in self-confidence for biomechanics because of TOPday. In group B, 20 students (87 %) and in Group C, 38 students (93 %) showed an increase in self-confidence (*p* > 0.05).

Fifty-one of the 64 students (80 %) who actively participated (Groups B and C) indicated that their enthusiasm for biomechanics increased because of TOPday. In Group B, 17 students (74 %) and in group C, 34 students (83 %) demonstrated an increase in enthusiasm (*p* > 0.05).

### Test results

**Fig. 4:**
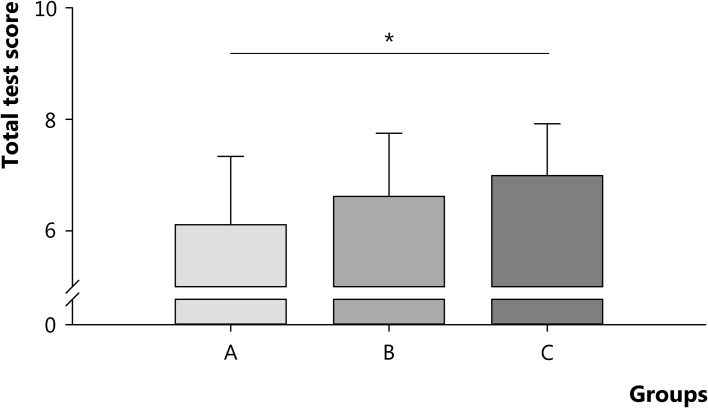
Total test score of the final course examination for groups A (*N* = 14), B (*N* = 23), and C (*N* = 41). Students in group A, B, and C completed 0–6, 7–18, and 19–24 questions, respectively. Data are presented as mean ± SD per group; * *p* < 0.05

The total test score of the final course examination was significantly different between the groups. Students in group A, B, and C scored a 6.1 (95 % CI 5.4–6.8), 6.6 (95 % CI 6.1–7.1), and 7.0 (95 % CI 6.7–7.3), respectively (Fig. 4). Students in group C scored higher than students in group A (*p* < 0.05) (Fig. 4).

**Fig. 5:**
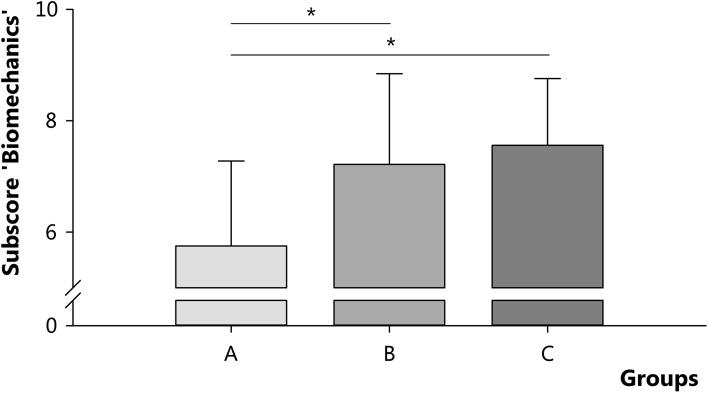
Subscore for biomechanics for groups A (*N* = 14), B (*N* = 23), and C (*N* = 41). Students in group A, B, and C completed 0–6, 7–18, and 19–24 questions, respectively. Data are presented as mean ± SD per group; * *p* < 0.01

When the subscore for biomechanics was analyzed, students in both groups B and C scored higher than students in group A (*p* < 0.01) (Fig. 5). Additional analyses demonstrated that students in group B and C scored significantly higher for biomechanics than for their total course examination (B: subscore 7.2 (95 % CI 6.5–7.9) vs. total-score 6.6 (95 % CI 6.1–7.1), *p* = 0.01; C: subscore 7.6 (95 % CI 7.2–7.9) versus total-score 7.0 (95 % CI 6.7–7.3), *p* = 0.002), whereas this effect was not present for students in group A (subscore 5.8 (95 % CI 4.9–6.6) versus total-score 6.1 (95 % CI 5.4–6.8), *p* = 0.2).

Students in group A, B, and C who had difficulties understanding mathematics and physics (*n* = 19) scored a 4.3 (95 % CI −5.3–13.8), 7.1 (95 % CI 5.4–8.9), and 7.2 (95 % CI 6.4–8.0), respectively. Students in group C scored significantly higher than students in group A (*p* < 0.05). Students in group A, B, and C who indicated mathematics and/or physics to be easy (*n* = 59) scored a 6.0 (95 % CI 5.1–6.9), 7.2 (95 % CI 6.4–8.1), and 7.7 (95 % CI 7.3–8.2), respectively. Students in group C scored significantly higher than students in group A (*p* < 0.01).

## Discussion

Many students in Biomedical Sciences have difficulty understanding biomechanics. Knowledge is retained longer if the subject material is repeated. In this study we tried to encourage students to repeat the subject matter by developing TOPday. TOPday consisted of multiple-choice questions on biomechanics with immediate feedback, which were sent via e-mail. We investigated the effect of TOPday on self-confidence, enthusiasm, and test results for biomechanics and showed that all of these were positively associated.

Sixty-four of the 78 students, i.e. 82 %, actively participated in TOPday. Students appreciated the new learning method ‘TOPday’. Questions were at a basic level, increasing in difficulty, and could be completed within a few minutes to facilitate the accessibility and persuade students to participate. Students stated that they would like ‘TOPday’ in other courses as well. Even the ones who hardly participated (Group A) mentioned that TOPday is a good methodology and that they were pleased by it. Some students, however, indicated that the questions were too easy for them; they would have preferred more difficult questions.

A significant number of students demonstrated an increase in self-confidence and enthusiasm for biomechanics due to ‘TOPday’. The increase in self-confidence and enthusiasm is probably related to the low key self-testing method of TOPday with immediate feedback. Although it was not asked to the students, it can be assumed that the students did not spend less time on the other study parts because of TOPday; in only a few minutes per day, students were able to rehearse biomechanics. TOPday seems, therefore, an effective way to encourage students to repeat the subject material, with the extra advantage that students are stimulated to keep on practising for the examination.

Although we demonstrated that students with an active participation in TOPday received higher grades for the final course examination, it can be questioned whether this was triggered solely by TOPday. It can be argued that the active students would score higher on biomechanics anyway. However, when the subscore for biomechanics was compared with the total score, it was shown that students in group B and C scored significantly higher for biomechanics than for their total course examination, whereas this effect was not present for students in group A. This suggests a positive effect of TOPday on the biomechanical test results. In future studies, this finding needs to be confirmed using multiple student cohorts. In addition, results of earlier study phases should be taken into account as well.

Based on the active participation rate of 82 %, we consider TOPday to be a successful method to motivate students for the subject matter. In future studies, it is recommended to develop questions with a higher difficulty to challenge the more excellent students. To further increase the participation level, it might be beneficial to develop an app for smart phones.

## Conclusion

The teaching method ‘TOPday’ seems an effective way to encourage students to repeat the subject material, with the extra advantage that students are stimulated to keep on practising for the examination. The appreciation was high. There was a positive association between active participation, on the one hand, and self-confidence, enthusiasm, and test results for biomechanics on the other. These results will be used to encourage students even more to participate actively in TOPday. In conclusion, students who actively participated in TOPday also had a TOPday during the examination.

## Essentials

- To improve the quality of medical education, a stimulating environment should be developed, in which students are actively involved in their own education.
- In this study, we developed (TOPday) consisting of multiple-choice questions on biomechanics with immediate feedback, which were sent via e-mail.
- The effect of TOPday on self-confidence, enthusiasm, and test results for biomechanics was studied.
- The appreciation for TOPday was high and 82 % of the students actively participated.
- There was a positive association between active participation, on the one hand, and self-confidence, enthusiasm, and test results for biomechanics on the other.^1^

## Appendix

See Appendix Table 1.

## Footnotes

1 The abstract of this article was published earlier as a short communication as a result of the author receiving the BSL prize.
